# Crystal Structure of the VapBC Toxin–Antitoxin Complex from *Shigella flexneri* Reveals a Hetero-Octameric DNA-Binding Assembly

**DOI:** 10.1016/j.jmb.2011.10.024

**Published:** 2011-12-16

**Authors:** Christian Dienemann, Andreas Bøggild, Kristoffer S. Winther, Kenn Gerdes, Ditlev E. Brodersen

**Affiliations:** 1 Centre for mRNP Biogenesis and Metabolism, Department of Molecular Biology and Genetics, Aarhus University, Gustav Wieds Vej 10C, DK-8000 Aarhus C, Denmark; 2 Centre for Bacterial Cell Biology, Institute for Cell and Molecular Biosciences, Newcastle University, Newcastle upon Tyne NE2 4AX, UK

**Keywords:** TA, toxin–antitoxin, PIN, PilT N-terminal, SIRAS, single isomorphous replacement with anomalous scattering, FEN-1, flap endonuclease-1, PDB, Protein Data Bank, DMSI, dimethyl superimidate, EMSA, electrophoretic mobility shift assay, RNA interferase, tRNA, protein–DNA interaction

## Abstract

Toxin–antitoxin (TA) loci are common in archaea and prokaryotes and allow cells to rapidly adapt to changing environmental conditions through release of active regulators of metabolism. Many toxins are endonucleases that target cellular mRNA and tRNAs, while the antitoxins tightly wrap around the toxins to inhibit them under normal circumstances. The antitoxins also bind to operators in the promoter regions of the cognate TA operon and thereby regulate transcription. For enteric *vapBC* TA loci, the VapC toxins specifically cleave tRNA^fMet^ and thus down-regulate protein synthesis. Here, we describe the crystal structure of the intact *Shigella flexneri* VapBC TA complex, determined to 2.7 Å resolution. Both in solution and in the crystal structure, four molecules of each protein combine to form a large and globular hetero-octameric assembly with SpoVT/AbrB-type DNA-binding domains at each end and a total molecular mass of about 100 kDa. The structure gives new insights into the inhibition of VapC toxins by VapB and provides the molecular basis for understanding transcriptional regulation through VapB dimerization.

## Graphical Abstract

**Figure:**
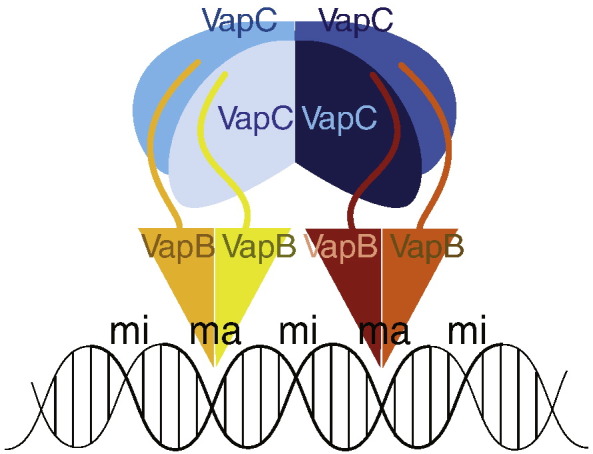

**Highlights:**

► *S. flexneri* VapBC form a large, 100-kDa hetero-octamer assembly. ► We observe two DNA-binding domains of the AbrB/SpoVT type. ► The complex binds tightly to operator DNA at two distinct sites. ► The structure suggests that induced fit takes place upon DNA binding.

## Introduction

Toxin–antitoxin (TA) loci are widespread in prokaryotes and code for an active “toxin” molecule, typically, a translational regulator, and an “antitoxin” that forms a tight complex with the toxin and thus inhibits it^1-3^. Upon changes in the surrounding environment, such as during nutritional stress, the antitoxin is degraded and the toxin is released intracellularly. Functions have not been ascribed to all types of toxins, but many possess “RNA interferase” activity, that is, they are able to cleave mRNA or tRNA to regulate overall rates of translation.^4^ At the genomic level, TA loci are organized in a tightly controlled operon with the toxin downstream of the antitoxin, transcriptionally regulated through a DNA-binding domain on the antitoxin.^5^ Type II TA loci, for which both toxin and antitoxin are proteins, have been subdivided into six evolutionarily independent families: *ccdAB*, *mazEF*, *phd/doc*, *parDE*, *higAB*, and *relBE*.^5^ VapC, HigA, and MazF toxins are active ribonucleases in isolation^3,6,7^ while the RelE-type toxins require the ribosome in order to cleave mRNA during translation.^8,9^ CcdB and ParE toxins, on the other hand, target DNA gyrase and thus inhibit DNA replication,^10,11^ while Doc has been proposed to inhibit translation through static binding to the ribosome in a way akin to antibiotics and thus does not induce RNA cleavage.^12^

In the largest TA family, *vapBC*, the VapC toxin contains a PilT N-terminal (PIN) domain, which is a compact ribonuclease domain consisting of an αβ-fold harboring four highly conserved acidic residues required for catalysis.^13^ PIN domains have only been functionally characterized in a few cases but are believed to cleave single-stranded RNA in a sequence and divalent metal-ion-dependent manner.^14^ They are found in all domains of life but are in bacteria mainly encoded by *vapBC* loci, where they are active on a range of substrates including mRNA and tRNA^14^ and often in a sequence-specific manner like their eukaryotic counterparts.^6^ Interestingly, *vapBC* loci are common among pathogenic bacteria, such as *Mycobacterium tuberculosis*, which contains an astounding 47 *vapBC* loci.^1^ The evolutionary benefit of having such an extreme number of similar genetic loci is not known, but recent results suggest that the loci are involved in the formation of persister cells, which are critical to pathogeniticy.^15^ Crystal structures of VapBC complexes and isolated VapC toxins currently exist from both archaea and the pathogenic bacteria *M. tuberculosis* and *Neisseria gonorrhoeae.*^16–18^ Like other TA systems, VapBC regulate their own transcription through binding of VapBC complexes to operators in its own promoter region.^19–21^ The structure of the *N. gonorrhoeae* FitAB complex, which is a VapBC-type TA system, bound to its operator site on DNA showed that the complex forms relatively loose hetero-octamer structure that interacts with DNA through two ribbon–helix–helix motifs.^18^ However, in the structure of unbound VapBC-5 from *M. tuberculosis*, the DNA-binding region of VapB was disordered and could not be resolved (Table 2).^17^

Recently, it was found that VapC (MvpT) from the Gram-negative pathogen *Shigella flexneri* 2a virulence plasmid pMYSH6000 functions by specifically cleaving initiator tRNA^fMet^ in the anticodon region, thus globally down-regulating translation.^22^ This showed that VapC toxins are capable of very specifically recognizing molecular targets and open up entirely new ways of fine-tuning cell metabolism. In order to understand the activity, mode of inhibition, and DNA-binding properties of the VapBC family, we have determined the crystal structure of the VapBC complex from *S. flexneri*. The structure reveals a compact hetero-octameric assembly with two unique DNA-binding domains of the SpoVT/AbrB type that have not been previously observed in VapBC complexes. Gel permeation chromatography and *in vitro* cross-linking experiments confirm that the octamer is present in solution and in the crystal, thus strongly suggesting that *S. flexneri* VapBC interacts with the promoter through interaction with adjacent major grooves.

## Results and Discussion

### Overall structure of the *S. flexneri* VapBC complex

His_6_-VapB:VapC was expressed in *Escherichia coli* from a bicistronic construct encoding genes optimized for expression, purified by Ni-NTA and gel-filtration chromatography, and concentrated to 7 mg/ml before crystallization. Large hexagonal crystals containing both components appeared in 1.0 M ammonium sulfate and 0.5% (v/v) polyethylene glycol 3350 at pH 5.5 and diffracted to about 2.7 Å. Following unsuccessful attempts at structure determination by molecular replacement using existing VapBC structures, the structure was eventually determined by single isomorphous replacement with anomalous scattering (SIRAS) using a uranyl acetate data set to 2.9 Å and refined to a final *R* (*R*_free_) of 18.2% (23.7%) using iterative rebuilding and refinement in Coot and PHENIX (see Table 1 for crystallographic data statistics). The asymmetric unit contains four copies of both VapB and VapC, for which the VapC structures are complete (residues 1–132), while the VapB structures cover residues 2–67 of the complete sequence (1–75). The C-terminal residues 68–75 of VapB extend into the solvent region and are not visible.

**Fig. 1:**
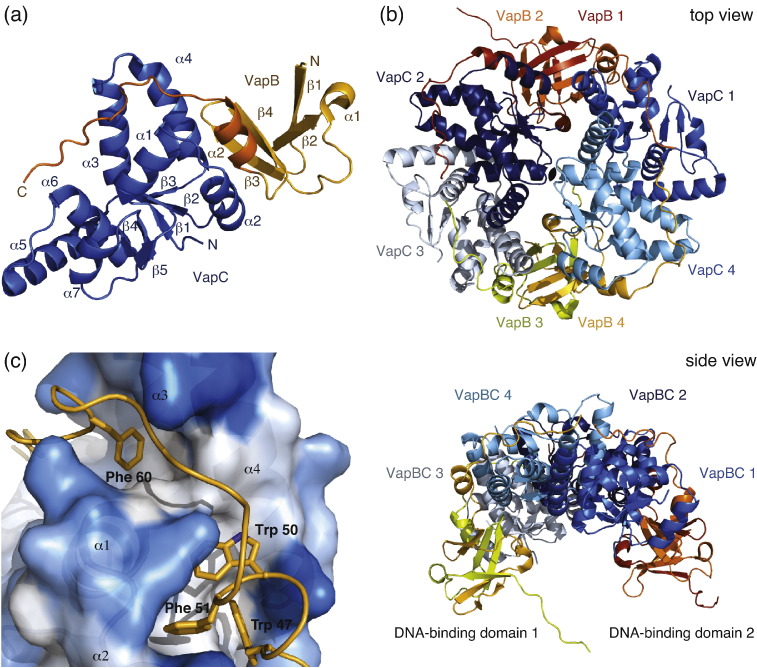
Structure overview. (a) The VapBC hetero-dimer shown in ribbon representation labeled with termini and secondary structure elements. The VapC toxin is in blue, and the VapB antitoxin is in orange. (b) Overview on the hetero-octameric assembly of four VapBC hetero-dimers found in the asymmetric unit, shown in top and side views with VapC in shades of blue and with VapB in shades of orange/yellow. The two DNA-binding domains formed upon dimerization of adjacent VapB molecules are orange and yellow, and on the top view, the dyad axis is indicated in a black lens shape. (c) Interaction of conserved hydrophobic residues in VapB (yellow) with the hydrophobic core of VapC. VapC is shown as a semitransparent surface colored from white (hydrophobic) to blue (hydrophilic) with the cartoon inside. This figure and subsequent structure figures were produced with PyMOL (Schrödinger, LLC).

*S. flexneri* VapC contains a typical PIN domain structure consisting of a small and central five-stranded β-sheet (β1–β5) surrounded by seven α-helices, denoted α1 through α7 (Fig. 1a, blue). VapB has an N-terminal domain consisting of four β-strands (residues 1–45), which dimerizes with a neighboring VapB to form a complete DNA-binding domain, and an extended C-terminal tail (residues 46–67) that wraps around VapC (Fig. 1a, orange). In the crystal, the four copies of VapBC in the asymmetric unit form a large and globular VapB_4_C_4_ hetero-octameric assembly, consisting of two VapB_2_C_2_ complexes related to each other by a dyad axis (Fig. 1b, top view; the dyad is indicated by the black lens shape at the center). The VapB_2_C_2_ complexes are strongly stabilized through interaction of the VapB N-terminal domains to form two DNA-binding domains (Fig. 1b, side view). The VapB_2_ dimer itself has an interface area of 1510 Å^2^ (Fig. 1b, orange/yellow chains) and consists of a layer of a four-stranded and a three-stranded antiparallel β-sheets. Interactions between the two VapC molecules in the VapB_2_C_2_ assembly are weak and water mediated (Fig. 1b, e.g., VapC_2_/VapC_1_, the dark-blue chains); however, there are strong interactions to the two VapC molecules of the adjacent VapB_2_C_2_ hetero-tetramer mediated through helices α4, α5, and α6 with a total interface area of 1050 Å^2^ (Fig. 1b, e.g., VapC_2_/VapC_3_, the dark-blue and white chains). Overall, these interactions give rise to a ring-shaped structure with weaker water-mediated contacts at the center. Interestingly, the DNA-bound conformation of the *N. gonorrhoeae* FitAB hetero-octamer shows a ring-shaped structure similar to but much more loose and open than that observed for VapBC (Table 2 and Supplementary Fig. 1).

### The VapB C-terminal domain inhibits VapC by a conserved mechanism

**Fig. 2:**
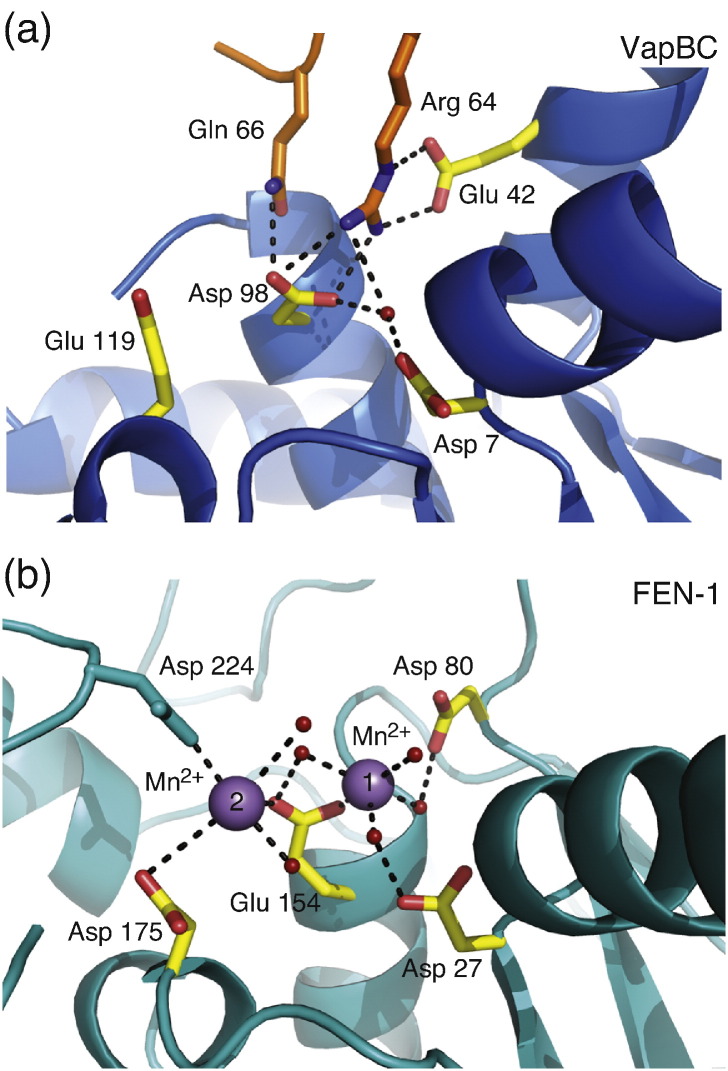
Comparison of the active site of VapC to FEN-1 nuclease. (a) Close-up of the active site of VapC shown in blue with yellow sticks and with relevant residues of VapB in orange sticks. Residues shown with yellow sticks are conserved in PIN domains. Water molecules in the active site are shown with red small spheres. (b) The active site of the *M. jannaschii* flap endonuclease (FEN-1; PDB 1A76^18^) shown in the same orientation as in (a). Conserved active-site residues are shown as yellow sticks; non-conserved residues, in green; and the two Mn^2^^+^ ions, as violet big spheres.

The interaction between the extended C-terminal region of VapB and the VapC PIN domain shows two main features, which are replicated in all four copies in the asymmetric unit. Firstly, four aromatic residues in VapB (Trp47, Trp50, Phe51, and Phe60) point into the hydrophobic interface between helices α1, α2, α3, and α4 of VapC where they interact tightly with the hydrophobic core of the protein (Fig. 1c). Secondly, near the C-terminus of VapB, the side chains of Arg64 and Gln66 of VapB point directly into the active site of VapC, where they make close, charged interactions to the conserved acidic active residues Asp7, Glu42, and Asp98 (Fig. 2a). In this interaction, the guadinium group of Arg64 interacts strongly with the carboxylic oxygen atoms of Glu42 and Asp98 in VapC through two hydrogen bonds to each residue. VapB Arg64 makes additional contacts to a water molecule located in the active site, which is also coordinated by Asp7, Asp98, and the main-chain nitrogen of Thr8 in VapC (the latter is not shown). In the FitAB complex, Arg68 in Fit B engages in a similar close interaction with the active-site residues of FitA, confirming the generality of this observation.^18^ In addition, a BLAST search using only the C-terminus of VapB (residues 46–75) identified 110 VapB proteins with a sequence similarity of 54–95%, and a multiple sequence alignment reveals a high degree of conservation for both interaction regions (i.e., the intercalating aromatic residues, as well as Arg64 and Gln66; Supplementary Fig. 2, marked in red). The high level of conservation strongly suggests that these are general mechanisms of inhibition within the VapBC group. Interestingly, however, in the structure of the *M. tuberculosis* VapBC-5 complex, the arginine was found in a different orientation, which may indicate that not all interactions are simultaneously required for inhibition to take place.^17^

In order to understand the functional implications of the close interactions at the active site, we superimposed the structure of VapB-inhibited VapC on the PIN domain from *Methanococcus jannaschii* flap endonuclease-1 [FEN-1; Protein Data Bank (PDB) ID: 1A76], which is more well characterized functionally.^23^ Overall, the superposition gives a good fit of secondary structure elements surrounding the active site and places all four conserved acidic residues in reasonable positions (Table 2 and Fig. 2b). Although the cleavage mechanism employed by PIN domains is still debated,^14^ there is growing evidence that it involves two divalent metal ions at the active site, coordinated by the acidic residues.^13,14^ Mutant studies implicate metal ion 1 (Fig. 2b, right) directly in the cleavage mechanism, whereas metal ion 2 (left) is thought to stabilize the conformation of the active site and enhance substrate binding.^23^ Both ions are present in the FEN-1 structure, and comparison to VapC reveals that the positively charged guadinium group of Arg64 in VapB appears to take up the position of metal ion 1 (Fig. 2b), while Gln66 may substitute metal ion 2. Together, this suggests that the nuclease activity of VapC is at least in part inhibited through displacement of the natural divalent metal ions from the active site.

### *S. flexneri* VapBC forms a higher-order structure in solution

**Fig. 3:**
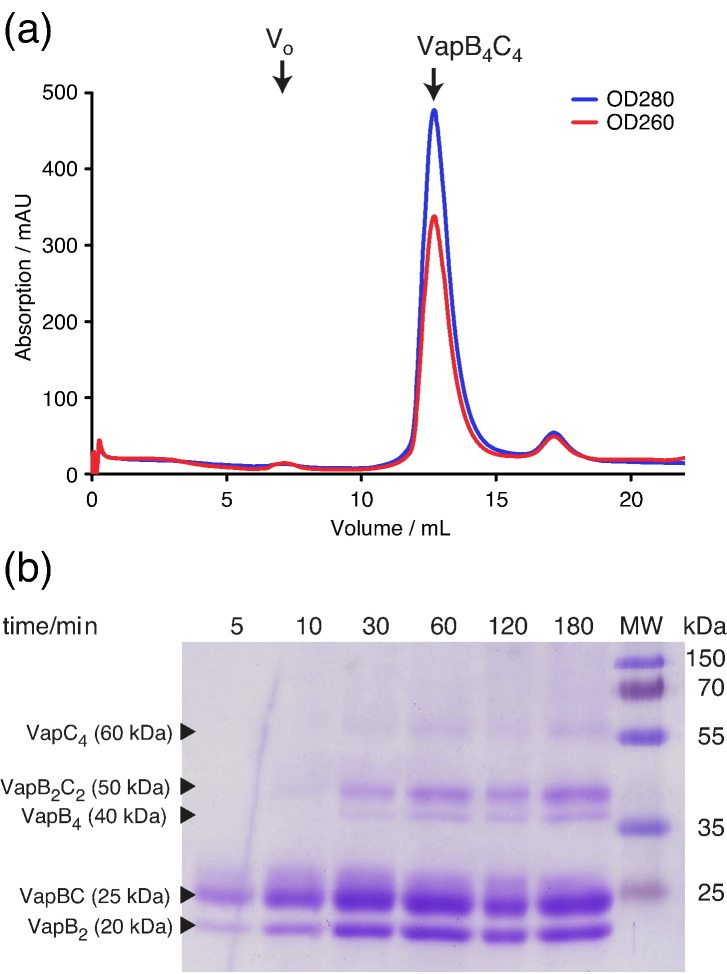
*S. flexneri* VapBC forms a hetero-octamer in solution. (a) Chromatogram from gel permeation chromatography using a 24-ml Superdex 200 10/300 GL column showing a major early peak containing both VapB and VapC at an elution volume of 12.7 ml, corresponding to a molecular mass of 105 kDa. *V*_o_ is the void volume (approximately 8 ml). OD_280_ is shown in blue, and OD_260_ is shown in red. (b) DMSI cross-linking time-course experiment analyzed by 15% Coomassie-stained SDS-PAGE with tentative assignment of the cross-linked species. MW is a molecular mass standard with the indicated sizes in kilodaltons. Monomeric VapC weighs 15 kDa, and VapB, 10 kDa.

As the higher-order octameric structure observed in the crystal structure of *S. flexneri* VapBC could arise due to crystal packing interactions, we asked if this VapBC complex also forms a higher-order structure in solution. Analysis of purified VapBC by high-resolution gel permeation chromatography using a 24-ml Superdex 200 10/300 GL column showed a major early peak containing both VapB and VapC around 12.7 ml. Consistently, this elution volume corresponds to a molecular mass of approximately 105 kDa (Fig. 3a), closely fitting the predicted size of a hetero-octameric VapB_4_C_4_ complex (VapC = 15 kDa, VapB = 10 kDa, and VapB_4_C_4_ = 100 kDa). We also analyzed the purified VapBC complex by chemical cross-linking using dimethyl superimidate (DMSI), which shows an increasingly complex pattern of higher-order complexes appearing over time (Fig. 3b). Early cross-linked complexes indicate formation of VapB_2_ (20 kDa) and VapBC (25 kDa) dimers (10 min), while later on, presumably due to variations in accessibility of cross-linking sites, VapB_4_, VapB_2_C_2_, and VapC_4_ species appear. The observation of a molecular species heavier than 50 kDa strongly indicates that a tetramer of VapC molecules is present in solution (molecular mass, 15 kDa) and, consequently, that the hetero-octamer is the prevalent oligomeric assembly due to the strong interaction between VapB and VapC. The lower abundance of the higher-order complexes on the gel is likely due to the statistical chance of cross-linking all four molecules in the same complex combined with accessibility of cross-linking sites. Thus, based on combination of gel permeation chromatography and chemical cross-linking, we therefore conclude that VapB and VapC assemble as an octameric complex in solution, consistent with the crystal structure.

### VapB dimerizes to form a SpoVT/AbrB-like DNA-binding domain

**Fig. 4:**
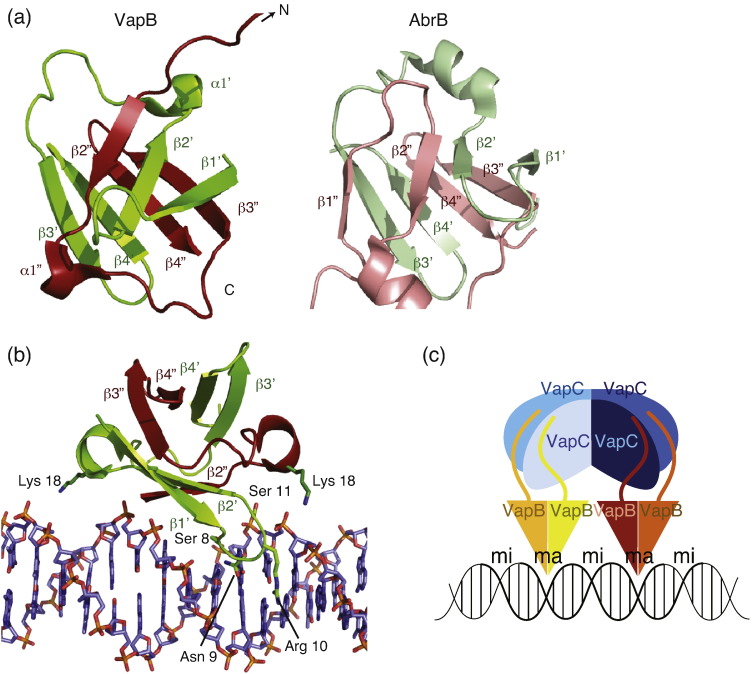
VapBC contains a SpoVT/AbrB-type DNA-binding domain. (a) Left: The DNA-binding domain in VapB resulting from homodimerization of two N-terminal domains (shown in red and green). Right: The homologous DNA-binding domain found in *B. subtilis* AbrB (light red/light green; PDB 2K1N^26^). The annotation of secondary structure elements for VapB follows the standard used for AbrB. (b) A model for the interaction between the VapB DNA-binding domain and linear B-form DNA with putative interacting residues in green. Both Lys18 residues from the VapB dimer interact with the DNA backbone, while residues 8–11 are proposed to interact with the DNA major groove. (c) An overview model of how DNA binding to adjacent major grooves might be accomplished by the VapBC hetero-octamer. Color scheme as in Fig. 1b. ma, major groove; mi, minor groove.

A BLAST search using the N-terminal sequence of *S. flexneri* VapB (residues 1–45) identifies the region as a putative SpoVT/AbrB-like DNA-binding domain (Pfam accession number PF04014). AbrB is a transcriptional regulator found in *Bacillus subtilis*, which is involved in the regulation of more than 60 genes.^24^ The solution structure of the DNA-binding domain of AbrB revealed the so-called “swapped β-hairpin” fold, in which two molecules each containing two β-hairpins dimerize through interweaving of the hairpins, forming a layered β-sandwich (Fig. 4a, right).^24^ Analogously, in the octamer structure of VapBC, the four N-terminal VapB domains come together to form two DNA-binding domains each consisting of a three-stranded antiparallel β-sheet (β1′ + β2′ and β2″; Fig. 4a, left, top layer) and a four-stranded antiparallel β-sheet (β3′ + β4″ and β3″ + β4″; left, bottom layer). The two β-sheets pack tightly against each other in a layered fashion to form the complete DNA-binding domain. Because the three-stranded β-sheets are formed asymmetrically (i.e., with one strand from one molecule and two from the other), two of four VapB molecules have an N-terminal domain consisting of two long and two short β-strands, while the other VapB molecules in our structure contain two long and a single short β-strand. The N-termini that only contribute a single short β-strand are extended and engage in crystal contacts with other symmetry-related octamers in the crystal. We speculate that the complete β-sandwich is formed upon binding to DNA. Interestingly, the DNA-binding domain is reminiscent of the β-barrel found in the MazEF TA complex but without the continuous interstrand hydrogen bonds required to form a proper β-barrel.^25^ This is therefore, to our knowledge, the first observation of a layered DNA-binding domain in bacterial TA systems.

In the DNA-binding domain of AbrB, the layers of β-sheets are connected by short two-turn α-helices. In comparison to VapB, we find that these are much shorter (Table 2 and Fig. 4a). In addition, in AbrB, the loops connecting β1′ and β2′ corresponding to residues 8–11 in VapB have been shown to be involved directly in sequence readout in the major groove of DNA, while the loops connecting β2 and β3 were predicted to be involved in DNA backbone recognition.^26^ Due to the aforementioned asymmetry in the present VapB structure, we only observe one of the major groove-binding loops at each end of the octamer, again suggesting that this region may undergo induced fit upon binding DNA. However, using the structure of the N-terminal domain of AbrB bound to its operator (PDB entry 2K1N), we can get some idea of how VapB binds DNA.^26^ In this model, the two Lys18 of both VapB molecules in the DNA-binding dimer are located close to the DNA backbone and probably contribute general affinity through charge–charge interactions (Fig. 4b). Inside the major groove, Ser8, Asn9, Arg10, and Ser11 from the β-loop come very close to the DNA bases and are therefore likely involved in sequence readout. We predict that an analogous interaction mediated by the other VapB molecule takes place in the same major groove on the other side of the double helix. The residues involved in direct contacts are only partially conserved across species, but similar functional groups are found close by in the related sequences, suggesting that the loops are used to adapt the domain to varying DNA sequences (Supplementary Fig. 3). Considering the entire octamer structure and the orientation of the two DNA-binding domains relative to each other, we find that the distance does not match exactly an integer multiple of helical turns and further that the domains are rotated 56° with respect to one another. This supports the idea that induced fit takes place upon DNA binding, either through deformation (bending) of the DNA or through conformational changes in the protein (Fig. 4c). Furthermore, the relative orientation of the two domains suggests that the DNA is approached from two sides and thus clamped by the VapBC complex.

### VapBC binds to two operator sites

**Fig. 5:**
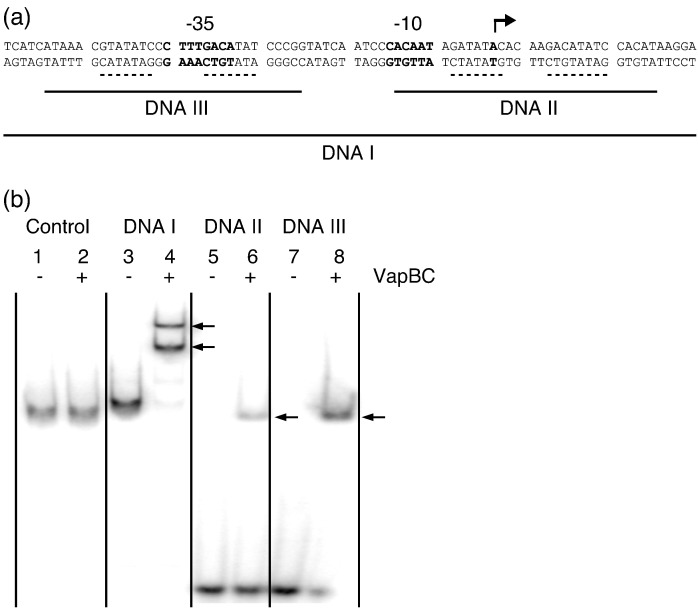
VapBC specifically binds the promoter DNA at two operator sites. (a) The sequence of the *S. flexneri vapBC* promoter region with the − 10 Pribnow box and the − 35 sequences is indicated in boldface. The transcription start site is shown with a bent arrow, and putative VapB binding sites in adjacent major grooves are indicated by broken lines. The position and extent of the DNA oligos (DNA I, DNA II, and DNA III) analyzed in (b) are indicated with continuous lines. (b) EMSA showing ^32^P-labeled DNA fragments binding to VapBC. Control DNA (lanes 1 and 2), DNA I (lanes 3 and 4), DNA II (lanes 5 and 6), or DNA III (lanes 7 and 8) were incubated with (+) or without (−) 2.5 ng/μl VapBC, and DNA and protein–DNA complexes were separated by 6% native PAGE. Arrows indicate shifted protein–DNA complexes.

To functionally investigate the binding of VapBC to promoter DNA, we first inspected the *vapBC* promoter region on the *S. flexneri* pMYSH6000 genome, which revealed two putative VapBC binding sites overlapping with the − 10 Pribnow box and the − 35 sequence (Fig. 5a, shown with broken lines). The specificity of VapBC toward these putative operator sites was then investigated by electrophoretic mobility shift assays (EMSA) using three different DNA fragments spanning either one or both of the regions. Incubation of the VapBC complex with the entire *vapBC* promoter region (DNA I) results in formation of two distinct complexes (Fig. 5b, lanes 3 and 4, and Supplementary Fig. 4a, lanes 7–12). VapBC is specific to these promoter sequences as no shifts are observed when the complex is incubated with an unrelated control DNA amplified from the pUC plasmid (Fig. 5b, lanes 1 and 2, and Supplementary Fig. 4a, lanes 1–6). This indicates that VapBC specifically binds to two sites in the *vapBC* promoter region. To further delineate the observed mode of binding, we incubated VapBC with two smaller DNA fragments containing the isolated operator sites (DNA II and DNA III; Fig. 5a and b). In each case, incubation produces single shifts (Fig. 5b, lanes 5–8). Since the VapB_4_C_4_ octamer is the stable form in solution, this strongly suggests that two VapBC octamers bind in the promoter region. Both DNA II and DNA III have identical lengths, and the shifts observed are of equal size, which indicates that the complexes formed with DNA II and DNA III are identical. However, the band shift observed for DNA III is of higher intensity than that for DNA II, which further suggests that VapBC has the highest affinity for the operator site overlapping with − 35 sequences (compare Fig. 5b, lanes 6 and 8, and Supplementary Fig. 4b, lanes 1–6 and 7–12). Both operator sites overlap with RNA polymerase binding sites; thus, VapBC binding to either site most likely results in transcriptional repression.

### Conclusion

In this paper, we have shown that the *S. flexneri* VapBC complex forms a large hetero-octameric assembly both in solution and in the crystal structure. This assembly most likely represents the biologically significant unit of the complex *in vivo*, and we predict that two copies of the assembly bind the promoter and thus regulate transcription from the VapBC operon, either through deformation of the DNA or protein induced fit. Previous crystal structures of isolated VapBC complexes from *N. gonorrhoeae*, *M. tuberculosis*, and archaeal homologues showed hetero-tetrameric multimers, while the DNA-bound structure of *N. gonorrhoeae* FitAB revealed a hetero-octameric assembly similar to that of *S. flexneri* VapBC, which seems to be required for DNA binding.^16–18^ However, FitAB contains a helical DNA-binding domain, and the structure is much more open than VapBC, which is the first example of a TA system containing an AbrB/SpoVT-type DNA-binding domain. The DNA-binding motif in VapBC forms through homodimerization of the antitoxin, and it may therefore be one of the main determinants for whether TA complexes form tetramers or octamers. Future studies will hopefully reveal the structural basis for promoter recognition by *S. flexneri* VapBC and the mode of interaction with the toxin target tRNA^fMet^.

## Materials and Methods

### Protein expression and purification

The construct pKW812HB^22^ was used for expression of His_6_-VapB:VapC in *E. coli* C41 (DE3). Cells grew in 1× LB containing 100 μg/ml ampicillin and were induced with 1 mM IPTG at an OD_600_ of 0.5. Expression was carried out overnight at 25 °C with vigorous shaking (120 rpm) before harvesting cells (15 min, 12,000*g*) and resuspension in 50 mM Tris (pH 8.0), 500 mM NaCl, 5 mM MgCl_2_, 5 mM β-mercaptoethanol, 10 mM imidazole, and protease inhibitor tablets (Sigma). For cell disruption, high-pressure homogenization at 15,000 psi and sonication were combined, and the resulting cell lysate was cleared by centrifugation at 15,000 rpm for 45 min and was loaded onto a pre-packed 5-ml HiTrap Ni-NTA column (GE Healthcare) followed by extensive column washing (20 column volumes) in 50 mM Tris (pH 8.0), 500 mM NaCl, 5 mM MgCl_2_, 5 mM β-mercaptoethanol, and 35 mM imidazole. Finally, the nearly pure VapBC complex was eluted with 50 mM Tris (pH 8.0), 500 mM NaCl, 5 mM MgCl_2_, 5 mM β-mercaptoethanol, and 300 mM imidazole. For crystallization and cross-linking, the sample was concentrated to 7 mg/ml using a Vivaspin 6 spin filter with a 5-kDa molecular mass cutoff and was further purified by gel filtration in 25 mM Tris (pH 8.0), 500 mM NaCl, 5 mM MgCl_2_, and 5 mM β-mercaptoethanol on a Superdex 200 10/300 HR column (GE Healthcare). VapBC used for EMSA was purified from *E. coli* C41 (DE3) containing the plasmid pKW812HC,^22^ which has the His-tag on VapC to prevent interference with the DNA-binding domain (VapB:His_6_-VapC). The protein was purified once by Ni-NTA using phosphate buffers [50 mM NaH_2_PO_4_ (pH 8.0), 0.3 M NaCl, 10/25/500 mM imidazole, and 5 mM β-mercaptoethanol] and subsequently dialyzed overnight against storage buffer (1× phosphate-buffered saline, 20% glycerol, and 1 mM DTT) at 4 °C.

### Chemical cross-linking

Prior to the experiment, the Tris buffer was exchanged for 100 mM sodium borate, pH 9.1, using a Vivaspin 6 spin filter with a 5-kDa molecular mass cutoff (GE Healthcare) to prevent reaction of the cross-linking agent with the free amines of the buffer molecules, and the protein was concentrated to 1 mg/ml. A reaction containing 50 μl of 1 mg/ml VapBC complex and 5 μl of 20 mg/ml DMSI was prepared, and11 μl aliquots was removed after 5, 10, 30, 60, and 120 min. The reaction was stopped with 4 μl of 2 M Tris, pH 8.0, and samples were analyzed by 15% Coomassie-stained SDS-PAGE.

### Crystallization and structure determination

For crystallization, the protein sample from gel filtration was concentrated to 7 mg/ml in gel-filtration buffer. Large single VapBC crystals appeared after 3–4 days in 0.1 M Bis-Tris (2-[bis(2-hydroxyethyl)amino]-2-(hydroxymethyl)propane-1,3-diol) (pH 5.5), 1 M ammonium sulfate, and 0.5% (v/v) polyethylene glycol 3350 at 19 °C using sitting-drop vapor diffusion in a 1:1 ratio of protein to crystallization buffer. Before data collection, crystals were cryo-protected by serial transfer into drops containing 10–20% glycerol and flash frozen in liquid nitrogen. Uranium derivative crystals were prepared by soaking the crystals for 3 h in mother liquor supplemented with 1.25 mM UO_2_(CH_3_OCOO^−^)_2_ (uranyl acetate). Data collection for native crystals was carried out at beamline ID23-1 at European Synchrotron Radiation Facility, Grenoble, with detectable diffraction to about 2.7 Å. The native data set has an unusually high redundancy (22-fold), giving rise to relatively high symmetry *R*-factors; however, the data are of very high quality, which are also witnessed by the redundancy-corrected *R*-factor *R*_mrdg,F_ (see Table 1). Uranium derivative crystals diffracted to 2.9 Å and data were collected at beamline i911-2 at MAX-Lab in Lund, Sweden. Indexing, integration, and scaling were carried out using XDS for both native and derivative data,^27^ and PHENIX was used to solve and refine the structure.^28^ Briefly, the uranium derivative data were used to solve the phase problem for VapBC by SIRAS. The initially obtained experimentally phased electron density map was submitted for automatic model building using phenix.autobuild, and the model was then iteratively updated manually and refined using Coot^29,30^ and phenix.refine, respectively.

### Electrophoretic mobility shift assay

The control DNA fragment and DNA I containing both *vapBC* operator sites were amplified in a PCR reaction using primers 171SR14 (5′-GGGGCAGCTGGCGAAAGGGGGATGTGCTGC) and 171SR16 (5-GGGGCAGCTGAATTTCACACAGGAAACAGCTA) and SF-EMSA-f (5′-GGCCGGCCCAGCGTTCTC) and SF-EMSA-r (5′-TGCTGAGAAATACGGTGG), respectively. Prior to the PCR amplification, primers 171SR14 and SF-EMSA-f were 5′ end-labeled with [γ-^32^P]ATP using T4 polynucleotide kinase (New England Biolabs). 171SR14 and 171SR16 produce a 199-bp DNA fragment of pUC plasmid DNA, while SF-EMSA-f and SF-EMSA-r produce a 212-bp DNA fragment containing the entire *vapBC* promoter region. The DNA II and DNA III fragments were created by hybridizing SF-EMSA1-f (5′-ACAATAGATATACACAAGACATATCCACAT) and SF-EMSA1-r (5′-ATGTGGATATGTCTTGTGTATATCTATTGT) and SF-EMSA2-f (5′-ATAAACGTATATCCCTTTGACATATCCCGG) and SF-EMSA2-r (5′-CCGGGATATGTCAAAGGGATATACGTTTAT), respectively. Prior to hybridization, primers SF-EMSA1-f and SF-EMSA2-f were 5′ end-labeled as described above. Labeled DNA fragments (1 nM) were incubated with purified protein complex in binding buffer [20 mM Tris–HCl (pH 7.5), 100 mM KCl, 2 mM MgCl_2_, 1 mM DTT, 50 μg/ml bovine serum albumin, and 10% glycerol]. To avoid nonspecific DNA binding, we added sonicated salmon sperm DNA to a final concentration of 0.1 mg/ml. Reactions were incubated for 20 min at 37 °C, protein–DNA complexes were separated by native PAGE in 6% acrylamide gels with 0.5× Tris–borate–ethylenediaminetetraacetic acid, and the separated complexes were visualized by phosphor imaging.

### Accession number

The structure of the *S. flexneri* VapBC complex has been deposited in the PDB with accession code 3TND.

## Figures and Tables

**Table 1:** Crystallographic data statistics

	Native	U (SIRAS)
*Data collection*		
Wavelength (Å)	0.94645	1.04002
Space group	*P*6_1_22	*P*6_1_22
Cell dimensions^a^		
*a*, *b*, *c* (Å)	91.4, 91.4, 549.1	92.4, 92.4, 548.9
α, β, γ (°)	90, 90, 120	90, 90, 120
Resolution (Å)	39.6–2.7	39.5–2.9
*I*/σ(*I*)^b^	20.5 (2.2)	28.76 (8.79)
Completeness (%)	99.6 (96.9)	99.5 (95.6)
Redundancy	22.4 (21.0)	6.1 (5.5)
Mosaicity (°)	0.1	0.09
*R*_sym_ (%)	16.6 (164.8)	4.3 (19.2)
*R*_mrdg,F_ (%)^c^	9.0 (70.7)	3.5 (18.1)

*Refinement*^d^		
Resolution (Å)	39.6–2.7	
No. of reflections	38,932	
*R*_work_/*R*_free_ (%)	18.2/23.7 (28.9/39.6)	
Total protein atoms	6280	
Total water molecules	205	
*B*-factor (Å^2^)	65.9	
RMSD bonds (Å)	0.0052	
RMSD angles (°)	0.838	
Ramachandran statistics^d^ (%)		
Favored	95.6	
Allowed	4.1	
Outliers	0.3	

Crystallographic data collection and refinement statistics for VapBC native and uranium derivative crystals.

a Values given by XSCALE.

b Figures in parentheses represent the outermost-resolution shell (2.76–2.69 Å).

c Redundancy-independent *R*-factor.

d Values given by PHENIX.

**Table 2:** Comparison of *S. flexneri* VapBC with similar structures

*Structural similarity (RMSD C^α^ positions) (Å)*		
VapC (core only)	*M. jannaschii* FEN-1 nuclease (PDB 1A76^23^)	3.44
VapC	*N. gonorrhoeae* FitB (PDB 2H1O^18^)	1.78
VapC	*M. tuberculosis* Rv0301 (VapC) (PDB 3H87)	2.15
VapC	*M. tuberculosis* VapC-5 (PDB 3DBO^17^)	0.97
VapB (N-domain)	*B. subtilis* AbrB (N domain, PDB 2K1N^26^)	3.66
VapB (β3 + 4 only)	*B. subtilis* AbrB (β3+4 only, PDB 2K1N^26^)	0.76

Root-mean-square deviation values (RMSD; measured in angstroms) are calculated by superpositioning of the indicated VapBC components (left column) onto other known structures (middle column). RMSD values are for C^α^ atoms only.
