# Supplementary material for: Crystal Structure of the VapBC Toxin–Antitoxin Complex from Shigella flexneri Reveals a Hetero-Octameric DNA-Binding Assembly
Source: J Mol Biol. 2011 Dec 16;414-540(5-7):713–22. doi: 10.1016/j.jmb.2011.10.024 (PMC3384007; doi:10.1016/j.jmb.2011.10.024)
Supplement: Supplementary file 1 — Supplementary materials [file mmc1.pdf]

Dienemann et al. Supplementary Figure 1

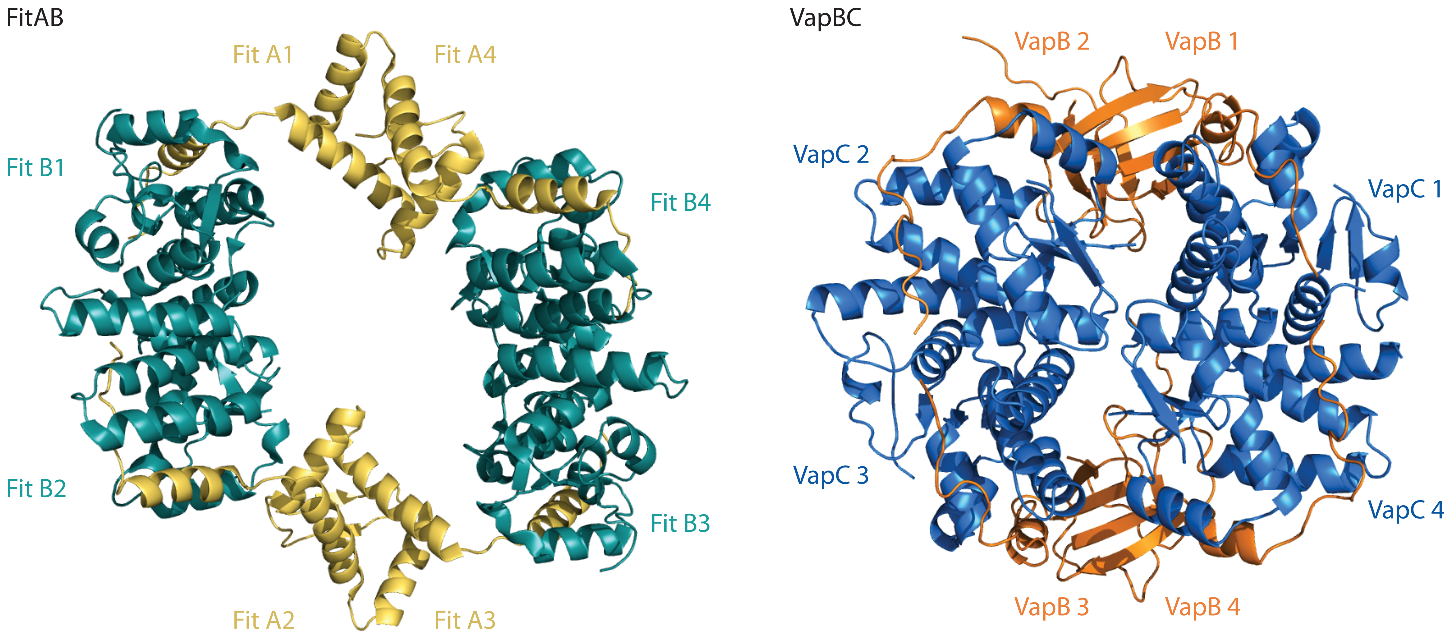

Supplementary Figure 1. Structural comparison of FitAB and VapBC. Left: The hetero-octameric structure of *N. gonorrhoeae* FitAB with the FitB toxin shown in sea green and the FitA antitoxin in yellow (PDB 2H1O[17]). The FitA antitoxins come together two-by-two to form two ribbon-helix-helix DNA binding domains (yellow, top and bottom). Right: The structure of hetero-octameric *S. flexneri* VapBC (this study) with the VapC toxin shown in blue and the VapB antitoxins in orange.

Dienemann et al. Supplementary Figure 2

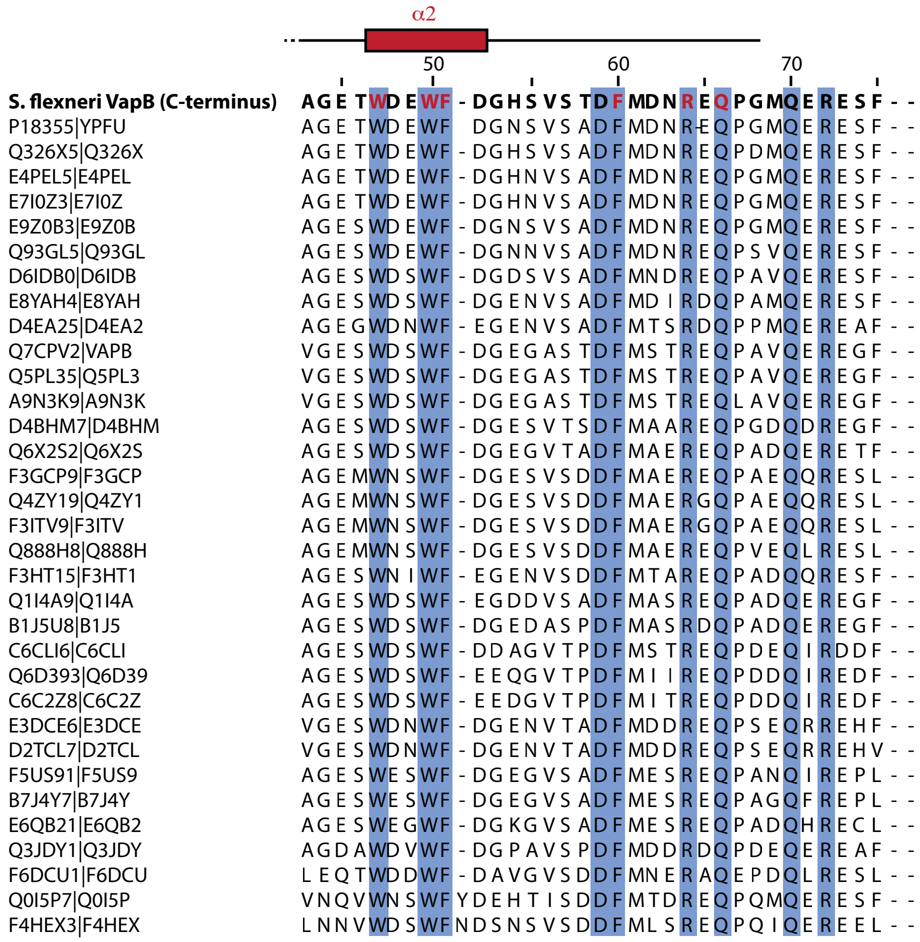

Supplementary Figure 2. Multiple sequence alignment of homologous VapB C-termini. Sequences with 54-95% identity to *S. flexneri* VapB are aligned and shown with their UniProt ID. Only the VapC-interacting region is shown corresponding to residues 43-75 of *S. flexneri* VapB. Conserved residues are marked in blue and residues directly involved in VapC inhibition in the structure presented here are shown with red letters in the first line. The secondary structure of VapB is shown at the top.

Dienemann et al. Supplementary Figure 3

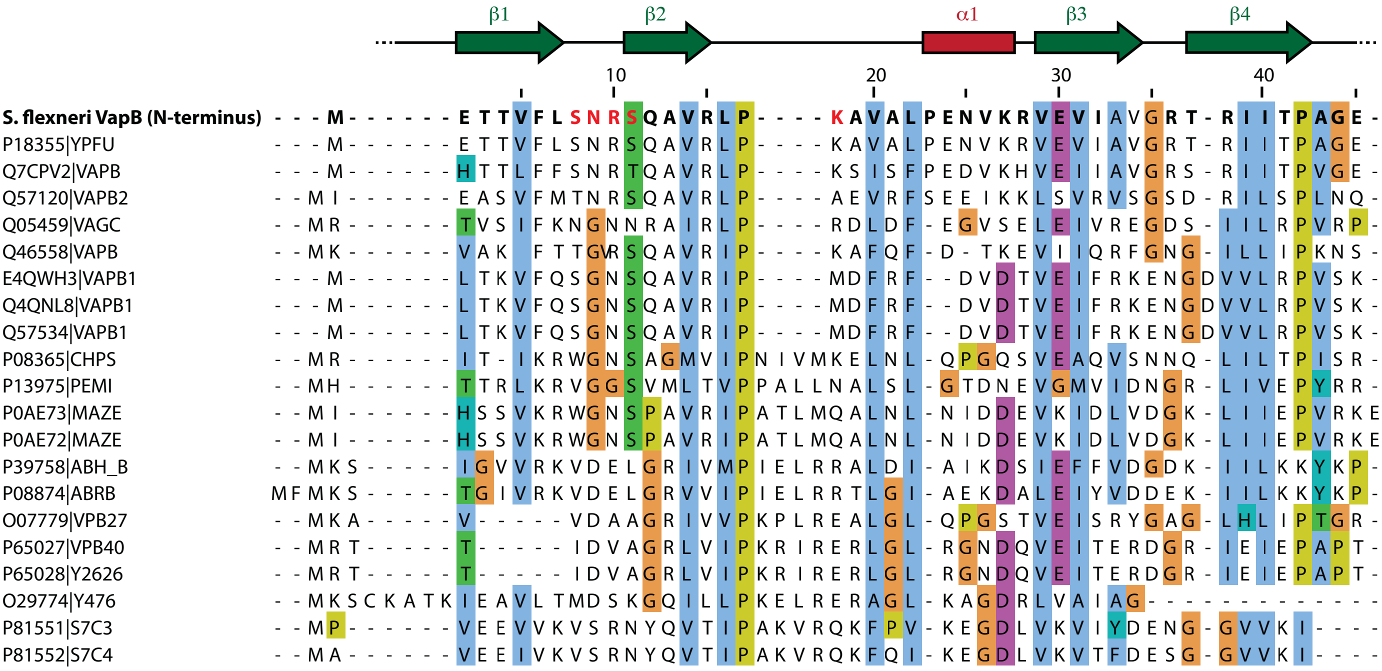

Supplementary Figure 3. Multiple sequence alignment of the VapB N-terminus with AbrB-like domains. Multiple sequence alignment of 22 proteins containing an AbrB-like DNA-binding domain showing conservation of several aliphatic residues (light blue), which tether the domain together. Acidic residues are shown on purple, hydrophobic (including His) on sea green, Ser/Thr on dark green, proline on light green, and glycine on orange background (Clustal colour scheme). The secondary structure of VapB is shown at the top.

Dienemann et al. Supplementary Figure 4

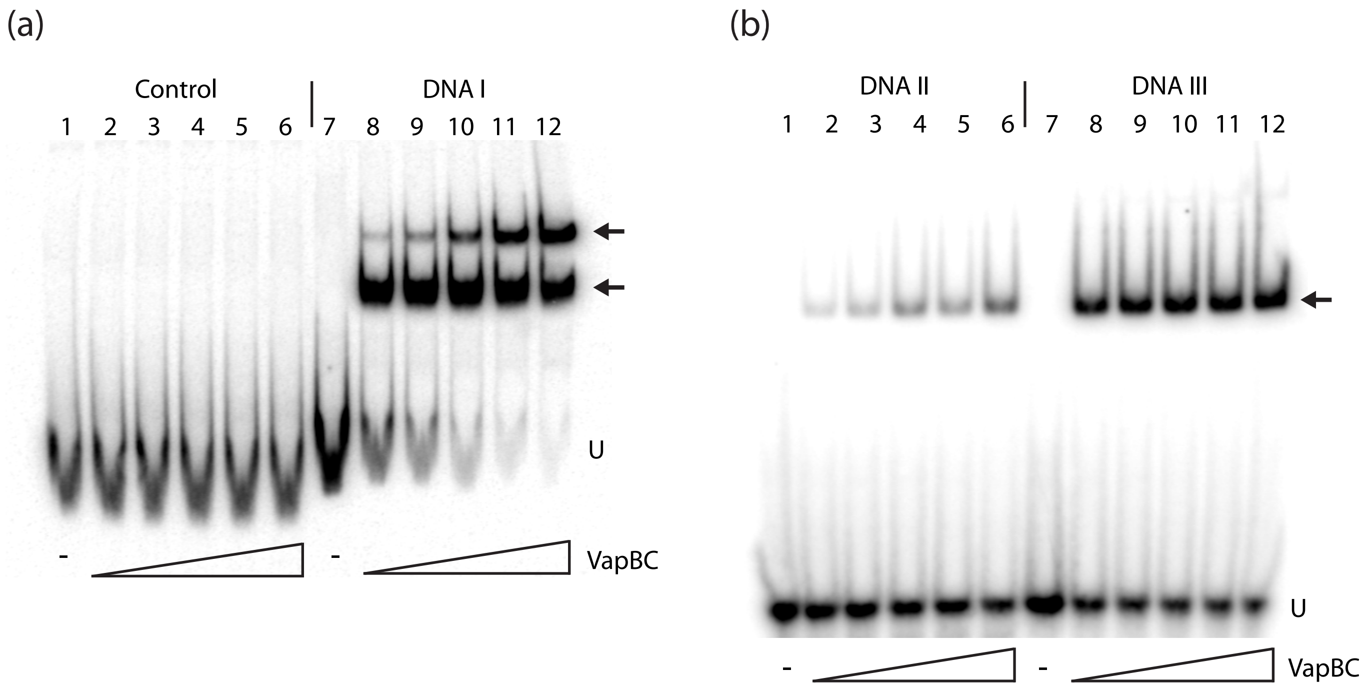

Supplementary Figure 4. Affinity measurements of the VapBC-DNA interaction. (a) Electrophoretic mobility shift assay (EMSA) with increasing concentrations of *S. flexneri* VapBC and constant amount of ^{32}P-labelled control DNA (lanes 1-6) or DNA I (lanes 7-14), see the Figure 5 legend for further details. DNA fragments were incubated with increasing concentrations of VapBC (0.2 - 2.5 ng/µL) and DNA-protein complexes separated by 6% native PAGE. ! indicates unbound DNA and arrows shifted DNA-protein complexes. (b) Electrophoretic mobility shift assay (EMSA) with purified VapBC and ^{32}P-labelled DNA II (lanes 1-6) or DNA III (lanes 7-14). DNA fragments were incubated with increasing concentrations of VapBC (0.5 - 4 ng/µL) and visualised as in (a).
